# Deep learning-based in-hospital mortality prediction using long-term sequential data in ICU patients: a multi-center validation study

**DOI:** 10.7717/peerj.21631

**Published:** 2026-08-24

**Authors:** Zihao Yan, Xueyu Wang, Xinran Xu, Yu Guo, Wei Chang, Jieyu He

**Affiliations:** School of Public Health, Kunming Medical College, Kunming, Yunnan, China

**Keywords:** Deep learning, Time-series information, Long-term survival, Stacked model

## Abstract

**Introduction:**

Intensive Care Unit (ICU) patients are at high risk of acute clinical deterioration and in-hospital mortality. Accurate prediction of in-hospital mortality is essential for optimizing clinical decision-making and resource allocation. However, most existing models rely primarily on short-term physiological data from a single hospitalization and lack adequate external validation. This study develops a deep learning-based model that integrates long-term temporal clinical sequences derived from patients’ historical ICU records with diagnostic information from the current ICU admission to predict in-hospital all-cause mortality during the current hospitalization. The proposed framework is applicable to both patients with a single ICU admission and those with multiple ICU admissions.

**Methods:**

We used current and historical International Classification of Diseases (ICD) codes, temporal features, and basic demographics to construct the prediction models. Single-admission patients were represented as one-event sequences, while repeated-admission patients were represented as longitudinal diagnostic trajectories. Model training and internal validation were conducted based on the MIMIC-IV database, while the eICU and MIMIC-III databases were used for external independent testing. Five deep learning (DL) models, including Informer and Transformer, were constructed for comparative analysis. On this basis, a stacked model integrating these five base models was further developed. The Area Under the Receiver Operating Characteristic Curve (AUROC), calibration curves, and clinical epidemiological analyses involving meta-analyses across different datasets were used to comprehensively evaluate the model’s predictive performance, generalizability, and specific clinical application value.

**Results:**

A total of 22,176 patients from Medical Information Mart for Intensive Care IV (MIMIC-IV), MIMIC-III, and eICU were included, and five deep learning models as well as one stacked ensemble model were constructed. In the internal validation set, the AUROC of most deep learning models exceeded 0.89, with the exception of the RNN. Among them, the Informer model achieved the best internal performance, with an AUROC of 0.95 (95% CI [0.928–0.967]). In the dual external validation across different databases, model performance exhibited apparent heterogeneity across cohorts. Although no single model consistently outperformed others, the stacked ensemble model showed relatively competitive overall predictive accuracy and stable generalizability.

**Conclusions:**

Compared with traditional models based on short-term biochemical indicators, the proposed deep learning models and the stacked ensemble model demonstrated competitive predictive performance for in-hospital mortality. This study demonstrates the potential of deep learning for ICU mortality prediction and proposes a novel framework for integrating longitudinal diagnostic trajectories into predictive modeling.

## Introduction

Intensive Care Unit (ICU) patients generally present with critical conditions and elevated mortality, with in-hospital mortality rates reaching 11%–42%. Notably, while ICU beds constitute just 10%–15% of a hospital’s total bed capacity, their operating costs make up 20%–25% of the institution’s overall expenses (Tang et al., 2022). Minimizing the mortality rate and medical costs of ICU patients, and rationally allocating ICU medical resources, are the primary challenges we currently face. The prediction of all-cause mortality in ICU patients is key to optimizing medical resource allocation and formulating personalized intervention plans. Over the past few decades, substantial research has been accumulated on survival prediction models for ICU patients. A range of assessment tools, including the Early Warning Score (EWS), Sequential Organ Failure Assessment (SOFA), Simplified Acute Physiology Score (SAPS), and Acute Physiology and Chronic Health Evaluation (APACHE) score, have been extensively applied in ICU clinical evaluations (Hwang et al., 2023). These models, built on early mortality prediction research, exhibit key limitations: first, their predicted mortality rates are consistently overestimated (Wang et al., 2023); second, they only provide ICU stay survival probability but fail to incorporate pre-admission diagnostic results or temporal diagnostic data, constraining predictive efficacy. At the algorithmic level, traditional linear combination models cannot synthesize diverse indicators to infer patients’ specific health status, and are thus ill-suited for predicting complex critical diseases such as Acute Kidney Injury (AKI) and Acute Kidney Failure (AKF) (George et al., 2021; Dong et al., 2021).

Machine learning (ML), especially its subfield deep learning (DL), has demonstrated significant advantages in interpreting complex signals in data-intensive environments (Alvi et al., 2023). The accumulation of large amounts of ICU clinical data, along with the accessibility of public datasets such as MIMIC-III, MIMIC-IV, and eICU, provides solid data support for the development of ML models in the field of critical care medicine. ML uses computational algorithms to learn from massive datasets, identify potential patterns and categories, and its core lies in training models with historical data to predict outcomes—serving as an alternative to traditional methods. The specific architecture of the stacking model has been detailed in the Appendix S1.

Currently, advanced DL models can leverage ICU monitoring data of patients to develop all-cause mortality prediction models, with the highest accuracy reaching 97.73% (Xie et al., 2023). Meanwhile, we have introduced a stacking model algorithm based on multiple DL models. A stacking model is an ensemble learning method: first, multiple base models independently perform predictions to generate their respective results; these results are then used as new features and input into a meta-model, which ultimately generates the final prediction. This method can better integrate the advantages of different models across various scenarios, achieving more excellent overall performance. Previous studies mainly focus on in-hospital mortality prediction and seldom incorporate longitudinal diagnostic trajectories available across ICU admissions into predictive models.

Although Electronic Health Records (EHRs) comprehensively capture diagnostic information from each ICU admission, few studies have fully leveraged these longitudinal diagnostic trajectories to predict in-hospital mortality among ICU patients (Xie et al., 2024; Molloy-Paolillo et al., 2023; Velez et al., 2025; Kim et al., 2023).

To address these limitations, this study uses existing public databases for model development and external independent validation. We developed and validated DL-based models (in particular, the Informer model proposed in recent years, which focuses on long-term sequence prediction, and the integrated stacking model) for prediction of in-hospital all-cause mortality in ICU patients. The proposed framework was designed to accommodate both patients with a single ICU admission and those with multiple ICU admissions by leveraging longitudinal diagnostic trajectories, diagnostic information from the current ICU admission, and demographic characteristics.

This method addresses the limitation that most existing mortality prediction models fail to fully utilize longitudinal diagnostic trajectories recorded across ICU admissions, thereby providing more comprehensive risk stratification for critically ill patients.

## Materials and Methods

**Ethical Considerations:** This study utilizes data from the Medical Information Mart for Intensive Care IV (MIMIC-IV) database. The research team has successfully completed the Collaborative Institutional Training Initiative (CITI) program examination and obtained official approval from the Institutional Review Boards (IRBs) of Beth Israel Deaconess Medical Center (BIDMC) and the Massachusetts Institute of Technology (MIT) to access and analyze the database (Johnson et al., 2023).

All patient data in the MIMIC-IV database have undergone rigorous de-identification processes and fully comply with the Safe Harbor provisions of the Health Insurance Portability and Accountability Act (HIPAA). Given the anonymized nature of the data and the IRB-approved exemption for studies using publicly accessible de-identified datasets, individual informed consent from research participants is not required (Shachar et al., 2023). This study was conducted in strict accordance with the ethical principles outlined in the Declaration of Helsinki (2024 version, https://www.wma.net/policies-post/wma-declaration-of-helsinki/), ensuring the protection of research participants’ rights, dignity, privacy, and confidentiality throughout the research process.

**Data Sources:** This study employed three public, de-identified critical care databases: MIMIC-IV (2008–2019, https://physionet.org/content/mimiciv/3.1/), a single-center repository containing 299,712 adult ICU stays from a U.S. tertiary academic medical center, which covers demographics, ICD codes, vital signs, laboratory data and clinical interventions; MIMIC-III (2001–2012, https://physionet.org/content/mimiciii/1.4/), an IRB-approved database including 46,520 ICU admissions from BIDMC, with longitudinal records such as demographics, vital signs, laboratory results and in-hospital mortality data; and eICU-CRD (2014–2015, https://eicu-crd.mit.edu/), a multi-center collaborative research database jointly developed by Philips Healthcare and the MIT Laboratory for Computational Physiology. It includes more than 200,000 ICU and step-down unit encounters across 208 hospitals in the United States, and its diverse multicenter population effectively enhances the generalizability and external validity of our established models (Lin et al., 2024; Hou et al., 2020; Kalimouttou et al., 2023).

**Figure 1:**
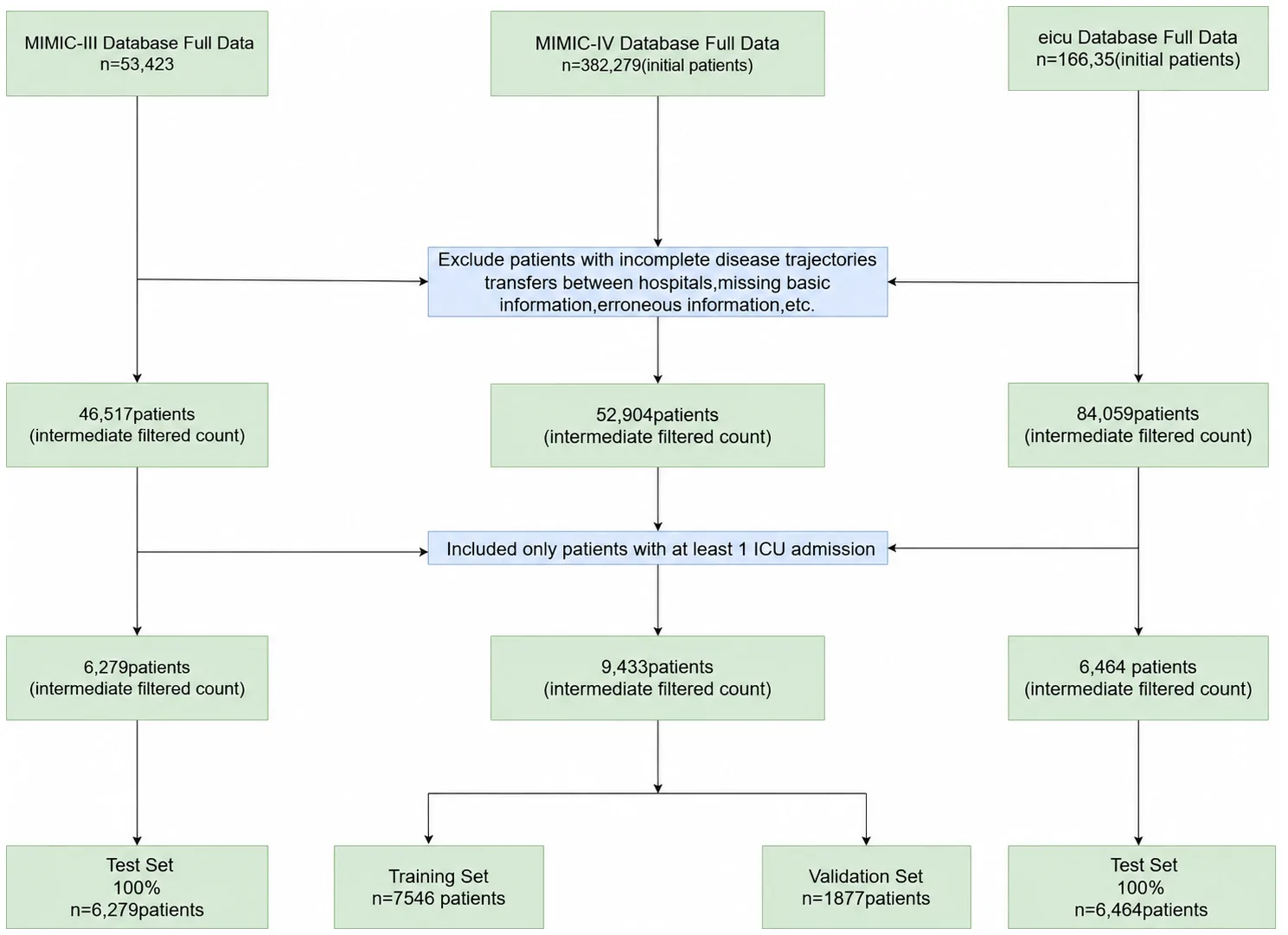
Inclusion and screening criteria for data.

**Inclusion Criteria**: We extracted ICU patient data from three databases (MIMIC-IV, MIMIC-III, and eICU) based on the following criteria: 1. Patients aged ≥18 years; 2. Patients with at least one documented ICU admission; 3. No inter-hospital transfer records (all ICU admissions occurred within the same hospital system); 4. No multiple ICU readmissions within a single calendar day. The detailed patient screening flow chart is shown in Fig. 1. Table 1 shows that a total of 9,433; 6,279 and 6,464 eligible patients were included from MIMIC-IV, MIMIC-III, and eICU, respectively.

**Table 1:** Baseline characteristics of patients in the dataset.

Characteristic	MIMIC-IV		MIMIC-III		eICU	
	All	Death	All	Death	All	Death
No. of participants	9,433	1,809 (19.1%)	6,279	1,235 (19.6%)	6,464	613 (9.5%)
Age (years), Median (P25–P75)	67 (56–78)	71 (60–81)	64 (53–75)	70 (58–79)	68 (57–79)	71 (60–79)
Gender *n* (%)						
Male	5,181 (55%)	1,008 (55.7%)	3,545 (53.5%)	715 (57.9%)	3,691 (57.1%)	348 (56.6%)
Female	4.252 (45%)	801 (44.2%)	2,734 (43.5%)	520 (42.1%)	2,773 (42.8%)	264 (43.3%)
Number of ICU admissions						
2	6,325 (67.0%)	1,117 (61,7%)	4,241 (68.0%)	765 (61.9%)	5,562 (86.0%)	507 (83%)
3	1,799 (19.0%)	384 (21.2%)	1,092 (17.4%)	240 (19.4%)	730 (11.3%)	84 (14%)
4	615 (6.5%)	145 (8.0%)	438 (6.98%)	111 (9.0%)	132 (2.0%)	14 (2.3%)
5	295 (3.1%)	74 (4.0%)	226 (3.60%)	54 (4.4%)	28 (0.4%)	4 (0.7%)
6	142 (2.2%)	31 (1.7%)	37 (1.5%)	26 (2.1%)	9 (<0.1%)	4 (0.7%)
7	85 (1.5%)	18 (0.9%)	46 (<1%)	13 (1.1%)	1 (<0.1%)	
8	49 (<0.1%)	6 (0.3%)	31 (<1%)	6 (<1%)	2 (<0.1%)	

**Disease Diagnosis Representation:** The patient diagnosis results are encoded using the International Classification of Diseases (ICD). For missing ICD codes, we apply masking layer technology to mask the missing data (Royen et al., 2021). To ensure consistency between datasets: all three databases have undergone the same preprocessing. The variables in the eICU dataset are consistent with the MIMIC data standard. Following the General Equivalence Mappings (GEMs) provided by the U.S. Centers for Disease Control and Prevention (CDC) and the Centers for Medicare & Medicaid Services (CMS), we converted synonymous codes from both datasets to the ICD-9 format, which accounts for a larger proportion of the data. For codes that could not be reliably mapped through the GEMs, we retained them in their original ICD-10 format. This approach maintains consistency between ICD-9 and ICD-10 codes while preserving clinical information that lacks direct equivalents. This standardization reduces biases that may be introduced by heterogeneous coding practices across databases.

**Prediction Target:** The prediction target of this study was in-hospital all-cause mortality during the current hospitalization. Model inputs included longitudinal ICD diagnostic trajectories derived from previous ICU admissions, diagnostic information recorded during the current ICU admission, demographic characteristics, and temporal intervals between admissions.

**Experimental Methods:** This study focuses on longitudinal time-series modeling of ICU patients with either single or multiple ICU admissions. By integrating clinical diagnostic data, time-series information, and demographic characteristics, a multi-dimensional deep learning framework is constructed. The specific experimental methods are as follows:

(1) Longitudinal data structuring

Integration of Data Dimensions and Construction of Time-Series Features: Multiple ICU admission records were arranged in chronological order to form individual-level time-series sequences. Using the patient’s unique identifier (subject_id), we constructed a three-dimensional matrix for each patient based on the structure of “time-set of diagnostic ICD codes—baseline characteristics”, incorporating information on multiple ICU admission times and ICD diagnostic codes for each admission. For example, the admission trajectory of Patient A is as follows: ([first admission time—set of ICD codes—baseline characteristics, second admission time—set of ICD codes—baseline characteristics, third admission time—set of ICD codes—baseline characteristics, …]). Core variables for each ICU admission were retained, including age, sex, ICD codes, and ICU admission time. Patients with multiple ICU admissions typically have multiple ICD diagnostic codes for each admission; these were integrated into a single variable to facilitate subsequent data processing (Placido et al., 2023). For patients with only one ICU admission, the same “time—set of diagnostic ICD codes—baseline characteristics” structure was retained, with the sequence consisting of a single admission event. Therefore, all patients, regardless of the number of ICU admissions, were represented using a unified longitudinal sequence format, ensuring consistent model input while preserving the complete admission history available for each individual.

(2) Temporal processing of diagnostic codes

To better utilize the temporal longitudinality of diagnostic records, for each patient, we incorporated the time record of their first ICU admission and the intervals between recorded admission times for each subsequent stay as model features. This was intended to enhance the temporal continuity and correlation of diagnostic data between consecutive ICU admissions. To capture longitudinal disease progression, previous ICU admissions for each patient were chronologically ordered. The time intervals between consecutive ICU admissions and the associated historical ICD diagnoses were extracted and encoded as structured predictor variables. These features were subsequently incorporated into the model together with demographic and clinical variables. Inter-admission intervals were computed and normalized as temporal features, which represent the difference (in days) between two consecutive admission times for the same patient, reflecting the interval between admissions. For patients with only one recorded ICU admission, the inter-admission interval was unavailable and was therefore recorded as missing. Meanwhile, the extracted temporal information was standardized using the *Z*-score normalization method to avoid the impact of excessive differences in feature scales and heterogeneity in time records between the training and external independent validation sets on model training. The standardized time-series information was then embedded into the models as new features, following the time information processing methods specific to each model.

(3) Balanced mini-batch sampling for class imbalance mitigation

Mortality was encoded as a binary label *y* ∈ {0,1}, where *y* = 0 indicates survival to hospital discharge and *y* = 1 indicates in-hospital death. Consistent with prior critical-care literature, pronounced class imbalance was present: observed mortality rates were 19.1% in MIMIC-IV, 19.6% in MIMIC-III, and 9.5% in eICU-CRD. Directly training on these skewed distributions induces a majority-class bias, wherein the model disproportionately optimizes for the prevalent survival class and exhibits markedly reduced sensitivity for the minority mortality class (He & Garcia, 2009). To address this limitation, we adopted a stratified mini-batch balanced sampling strategy. The complete dataset was first partitioned into two mutually exclusive strata—survivors (*y* = 0) and non-survivors (*y* = 1). The stratum with the smaller cardinality (non-survivors) was designated as the reference size. During each training iteration, we randomly sampled n instances from each stratum without replacement to construct a balanced mini-batch (*P* (*y* = 0) = *P* (*y* = 1) = 0.5). This procedure was repeated with reshuffling at the epoch level to ensure full utilization of all training instances while preserving class balance within every gradient update. By equalizing prior probabilities at the batch level, the proposed scheme effectively mitigated the deleterious effects of class imbalance across all three databases, thereby enhancing both the discriminative performance and calibration of the in-hospital mortality prediction models (Wang & Cheng, 2021; Zhang, Kjellström & Mandt, 2017).

(4) Prevention of model overfitting

The core objective of preventing overfitting is to avoid the model overlearning noise and idiosyncratic details in the training data, ensuring it maintains strong generalization ability on unseen new data. This enables the reliable identification of true patterns (rather than memorizing spurious features of training samples) in practical applications, thereby enhancing the model’s generalizability and credibility. We employed dropout, L2 regularization, and early stopping mechanisms to prevent model overfitting: Information embedding dropout: Randomly zeroing out ICD embedding vectors with a certain probability to enhance the model’s robustness to sparse diagnostic data. Regularization: L2 regularization was added to the recurrent layers of long short-term memory (LSTM)/gated recurrent unit (GRU)/ recurrent neural network (RNN) and the feed-forward networks of Transformer/Informer. Early stopping mechanism: Based on model evaluation on the internal validation set, training was terminated if no improvement was observed for 10 consecutive epochs to avoid overfitting (Srivastava et al., 2014; Zaremba, Sutskever & Vinyals, 2014; Prechelt, 1998).

(5) Dataset partitioning for model training, validation, and testing

We randomly split the MIMIC-IV data into training (80%) and validation (20%) sets. To ensure robust external validation, we used the entirety of MIMIC-III and eICU as independent external test sets to evaluate the model’s generalizability and performance when applied to unseen datasets.

(6) Hyperparameter optimization

To enhance model generalization, we conducted a grid search over key hyperparameters, including learning rate, number of layers, regularization coefficients, and other architecture-specific parameters (Tran, Le & Lam, 2022). For each model, we predefined reasonable search ranges for these hyperparameters. The grid search method systematically evaluated different combinations within these predefined ranges to identify the optimal hyperparameter configuration for maximizing predictive performance. The detailed grid search ranges for each individual model are provided in Appendix S1 (please refer to the appendix for specific numerical ranges and candidate values).

(7) Stacking model construction

Five architectures (Informer, Transformer, LSTM, GRU, RNN) and a stacked ensemble were trained using early stopping and class-balanced sampling. Using ICD diagnostic sequences as the core input, the model integrated structured features including time intervals, age, and gender. Training employed balanced sampling and early stopping, retaining optimal parameters from the validation set. The meta-learner, a Multilayer Perceptron (MLP), takes base models’ predictions and structured features as inputs. These inputs undergo dynamic weighting *via* independent linear layers, followed by concatenation to output the fused mortality risk probability. Optimized *via* early stopping to prevent overfitting, it enables complementary integration of base models’ strengths (Wang et al., 2025).

(8) Evaluate model performance on the validation set

External validation of the models trained and validated on MIMIC-IV was performed using eICU and MIMIC-III as external validation sets. The area under the receiver operating characteristic curve (AUROC) and The calibration curves were used as primary evaluation metrics. Meanwhile, a meta-analysis of risk groupings derived from model outputs across the two external datasets was conducted to comprehensively evaluate their application value, thereby effectively validating the models’ accuracy and utility (Tan et al., 2020; Brabrand, Hallas & Knudsen, 2014).

## Results

### Characteristics of the study participants

We extracted ICD diagnosis codes from patients with at least one ICU admission. These codes served as the primary features, along with three fixed categorical indicators: age, gender and number of ICU admissions. After applying the inclusion and exclusion criteria detailed in Fig. 1, we extracted all eligible patient records from the MIMIC-IV, MIMIC-III, and eICU databases. Among them, there were 9,433 participants in the MIMIC-IV database, of which 7,546 (80%) were assigned to the training set and 1,887 (20%) to the internal test set. Under the same inclusion and exclusion criteria, all 6,279 participants in the MIMIC-III database and 6,464 participants in the eICU database were included as independent external test sets to evaluate the deep learning model developed on the MIMIC-IV cohort. Table 1 presents baseline characteristics of filtered patients from the three datasets. Age and sex distributions varied minimally across databases, with age interquartile ranges (IQRs) in MIMIC-IV (56,67,78), MIMIC-III (53,64,73), and eICU (57,68,79), and male-to-female ratios ranging 1.23–1.33. Mortality rates were similar in MIMIC-IV (19.1%) and MIMIC-III (19.6%) but lower in eICU (9.5%).

Cochran-Armitage trend tests on multicenter data (MIMIC-IV, MIMIC-III, eICU) revealed a significant positive association between ICU admission frequency and mortality (all *P* < 0.05). In MIMIC-IV, mortality rose from 17.7% (two admissions) to 22.7% (≥5 admissions; *χ*^2^ = 23.03, *P* < 0.001). MIMIC-III showed a stronger trend (*χ*^2^ = 38.43, *P* < 0.001), with ≥5 admissions (29.1%) having higher mortality than two admissions (18.0%). Despite limited high-frequency admission samples, eICU also showed a significant trend (*χ*^2^ = 7.54, *P* = 0.006), with ≥5 admissions (20.0%) having 2.2-fold higher mortality than 2 admissions (9.1%).

### Development and validation of an in-hospital mortality prediction model

**Figure 2:**
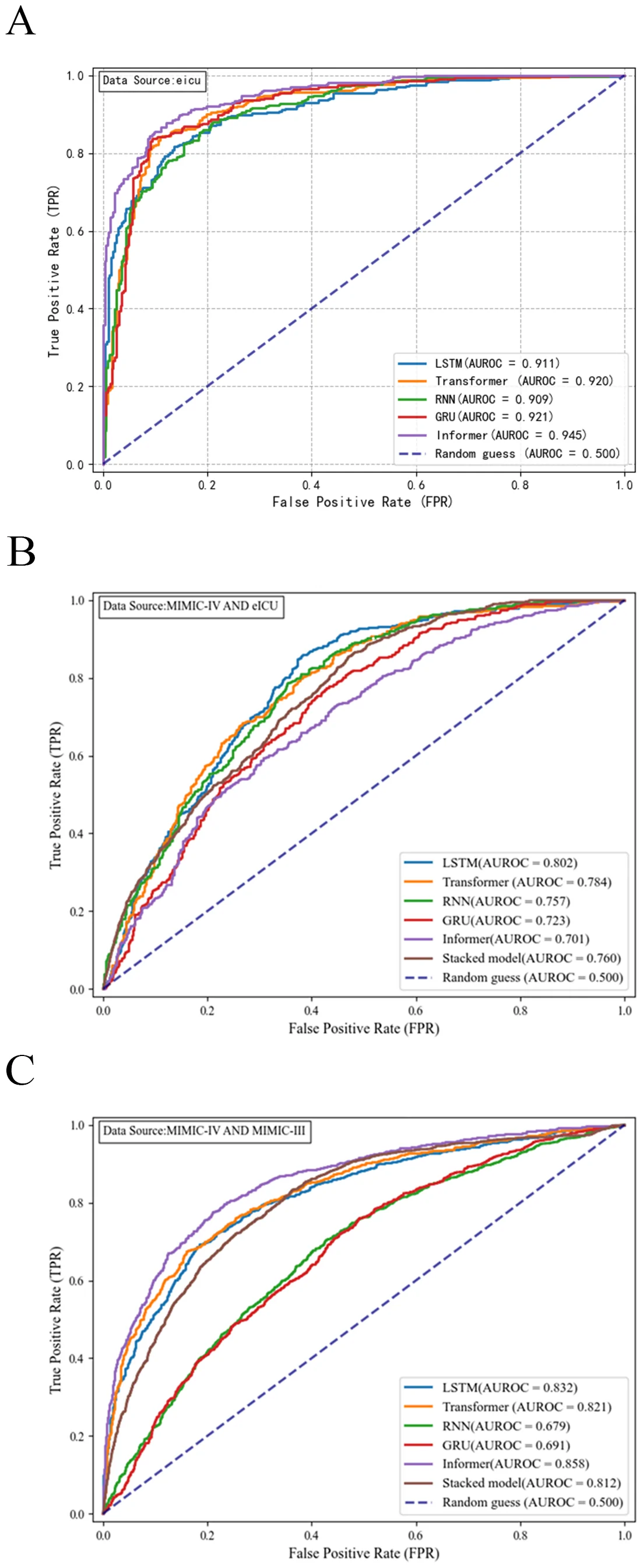
AUROC values of the models across different validation datasets. (A) Internal validation AUROC in the MIMIC-IV cohort; (B) External validation AUROC in the eICU cohort; (C) External validation AUROC in the MIMIC-III cohort.

This study used the MIMIC-IV database for model training and internal validation, and randomly split the data into a training set and a validation set at a 4:1 ratio. Model performance was evaluated by plotting the AUROC and calibration curves. We further calculated the Area Under the Precision–Recall Curve (AUPRC), F1-score, and recall to supplement and validate the stability of all models, with relevant results presented in the Appendix S1. For the stacking model, the base-model outputs were used as inputs to the meta-learner, and detailed descriptions of the stacking framework and dataset construction are provided in the Appendix S1. As shown in Fig. 2A, a comparison of the internal validation AUROCs of the five base models revealed that the AUROC of all models except the RNN exceeded 0.91, indicating good internal discrimination. Their internal validation performance supported subsequent external validation.

Subsequently, all models were externally validated in the eICU and MIMIC-III cohorts, with the AUROC comparison results shown in Figs. 2B and 2C, respectively. To reduce temporal heterogeneity during the evaluation of the eICU database, records in the MIMIC-IV database were re-screened to include only patients admitted within the past 2 years (the detailed exclusion criteria and methods are provided in the Appendix S1). Under this condition, the LSTM network achieved the highest AUROC of 0.802 (95% CI [0.774–0.828]), followed by the Transformer model and the stacking model—both of which exhibited strong discriminative ability. Although the AUROCs of the RNN, GRU, and Informer were relatively lower, they still fell within an acceptable range. This indicates that these models can provide meaningful prognostic signals and possess a certain degree of generalizability. However, compared with the internal validation cohort, model performance generally declined in the external validation cohorts, particularly in the eICU dataset, suggesting that predictive performance may be influenced by differences in patient populations and clinical settings.

**Figure 3:**
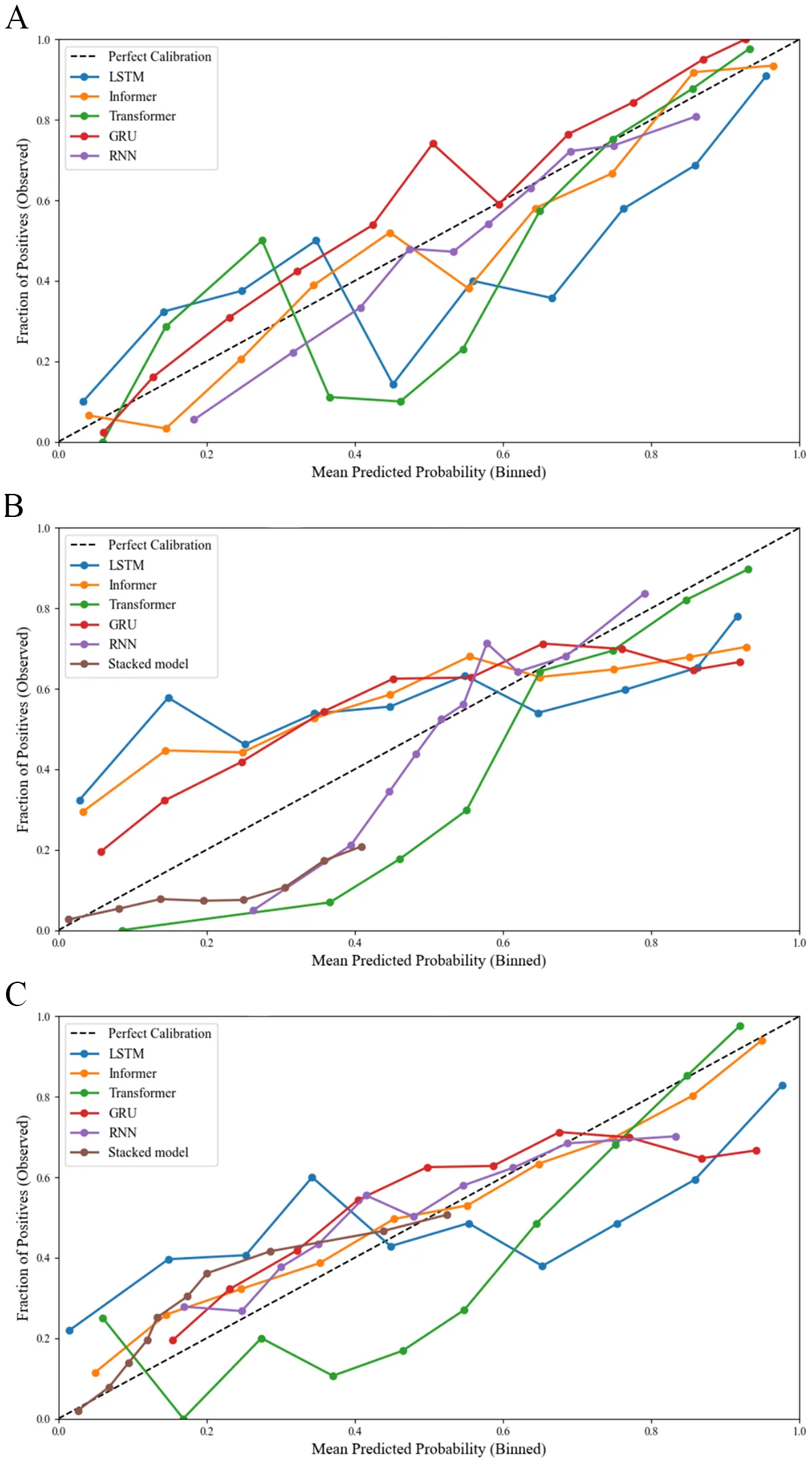
Calibration curves of the models across different validation datasets. (A) Calibration curves in the MIMIC-IV internal validation cohort; (B) Calibration curves in the eICU external validation cohort; (C) Calibration curves in the MIMIC-III external validation cohort.

In the MIMIC-III external cohort, the Informer model achieved an AUROC of 0.858 (95% CI [0.821–0.884]), outperforming the Transformer, LSTM, and stacking models; in contrast, the GRU and RNN showed relatively poor performance. Calibration curves from the MIMIC-IV, MIMIC-III, and eICU databases (Fig. 3) provided additional insights into model reliability. In the MIMIC-IV and MIMIC-III cohorts, the Informer model’s calibration curve lay close to the diagonal, indicating good agreement between predicted probabilities and observed event rates across most risk strata. Most other models (LSTM, Transformer, GRU, RNN) also showed reasonable calibration in these two internal-derived cohorts, though with slight deviations at higher predicted risks. In contrast, in the eICU cohort, the calibration curves of most models were not as closely aligned with the diagonal, particularly in the mid-range of predicted risks. Notably, the stacking model demonstrated relatively better calibration in the eICU cohort compared to the individual base models, with its curve lying closer to the ideal line. Regarding overall comprehensive performance across external validations, the stacking model showed relatively prominent discriminative ability (AUROC) on the two independent external test sets (MIMIC-III and eICU), reflecting its capacity to integrate the strengths of the five base models. However, no single model consistently outperformed others across all test sets. For instance, the Informer model achieved optimal AUROC in the MIMIC-III cohort (longer time span) but performed poorly in the eICU cohort (shorter time span), and its calibration also deteriorated in that external setting.

To explore the impact of patients’ ICU length of stay on model performance, this study evaluated models trained and validated on datasets partitioned by different time periods. Relevant results are provided in the Supplemental Information.

### Meta-analysis of stacking model predictions

**Figure 4:**
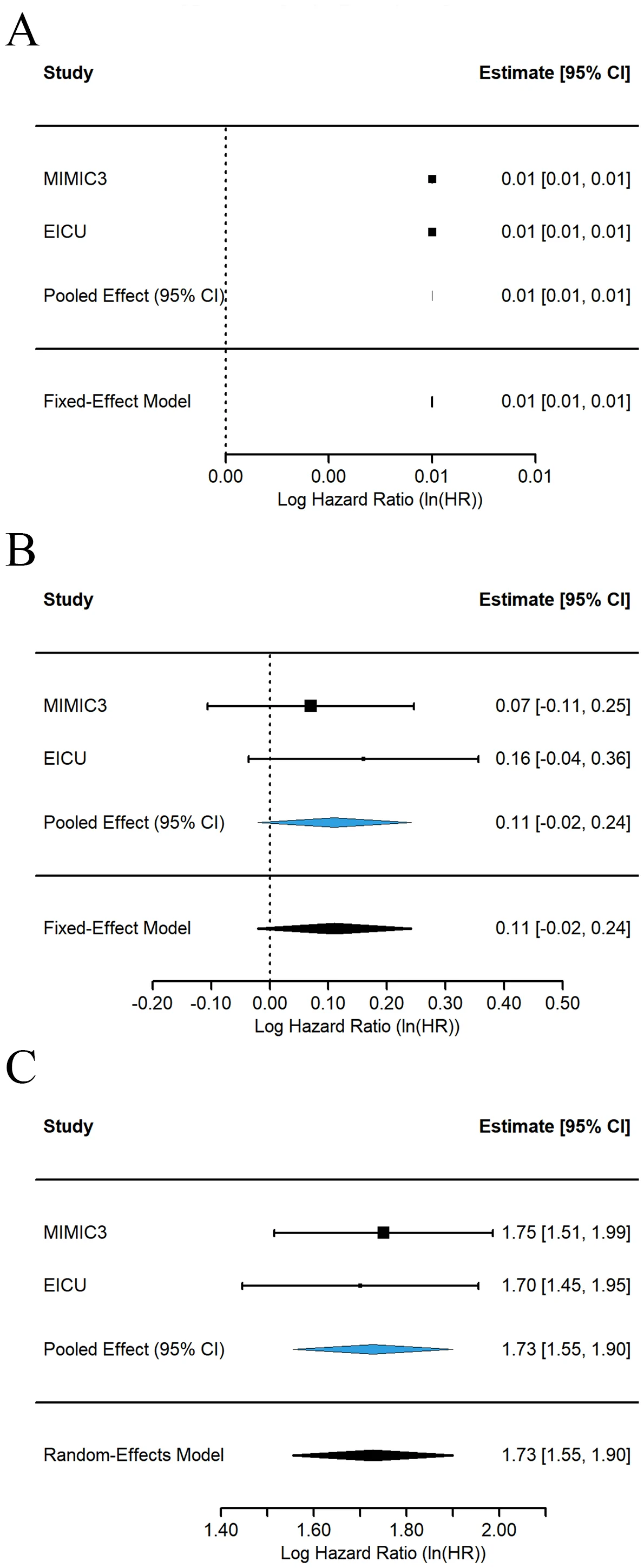
Meta-analysis of risk groups and baseline data. (A) Meta-analysis results of age; (B) Meta-analysis results of gender; (C) Meta-analysis results of risk group.

To further validate the prognostic value of the stacking model’s risk stratification, we conducted a meta-analysis of the model’s predictions. Patients were divided into high-risk and low-risk groups based on the model’s continuous risk score (derived from normalized time-to-event data), using the optimal cutoff determined by the Youden index in the training cohort and subsequently applied to all validation cohorts, with adjustment for baseline covariates (age and gender). Compared with the low-risk group, the high-risk group had a significantly increased risk of death: under the random-effects model, the natural logarithm of the hazard ratio [ln(HR)] was 1.73 (95% CI [1.55–1.90]), corresponding to a hazard ratio (HR) of approximately 5.65 (95% CI [4.71–6.69]), with *P* < 0.001. Figure 4 presents the forest plot of pooled HRs, clearly illustrating the significant mortality difference between the high-risk and low-risk groups across all included cohorts. The primary endpoint was 30-day mortality, with patients who survived beyond 30 days classified as survivors.

Subgroup analyses by age and gender showed that the relative risk of the high-risk group remained highly consistent across all strata, with negligible effects of age or gender. When age was included as a continuous covariate in the multivariate regression (fixed-effects model), the pooled estimate was ln(HR) = 0.01 (95% confidence interval [0.01–0.01]), indicating that each 1-year increase in age was associated with an approximately 1% increase in the risk of adverse outcomes, with minimal heterogeneity across cohorts. In the gender analysis, the comparison between males and females yielded ln(HR) = 0.11 (95% CI [−0.02–0.24]); the confidence interval included zero, indicating no statistical significance.

Overall, these results support the external validity and robustness of risk stratification based on the stacking model in critically ill populations. The model can effectively distinguish between high-risk and low-risk patients and provide prognostic information independent of age and gender. These findings suggest the potential utility of the model for clinical risk stratification, although further prospective validation is warranted before routine clinical implementation.

## Discussion

Accurate prediction of in-hospital mortality remains an important challenge in critical care. This study shows that deep learning models using only diagnostic and demographic data can achieve robust discrimination across three large databases. The advent of the big data era and the exponential improvement in ICU monitoring accuracy have given rise to large-scale databases of patients’ longitudinal information, providing fertile ground for the application of deep learning (DL) algorithms. Such algorithms can convert this data into accurate, actionable prediction results, with application scenarios ranging from disease diagnosis to mortality risk prediction (Perin et al., 2022). A core advantage of deep learning lies in its ability to independently identify potential nonlinear relationships and high-level feature representations from highly complex high-dimensional data (Rithani, Prasanna Kumar & Doss, 2023), without relying on traditional statistical probability calculations. This study applied deep learning to a comprehensive set of clinical variables, including not only age and gender but also time-structured information (*e.g.*, intervals between multiple ICU admissions, ICD diagnostic codes corresponding to each admission). Numerous previous studies have confirmed that the performance of deep learning architectures is generally superior to that of classical linear models and modern machine learning models such as Random Survival Forests (RSF) (Sze et al., 2017). Additionally, the framework proposed in this study incorporates in-hospital mortality outcomes and longitudinal diagnostic trajectories from previous ICU admissions, enabling diagnosis-informed risk stratification during the current hospitalization.

This study has several inherent limitations that should be acknowledged when interpreting its findings, as outlined below:

1. Dataset temporal scope and model architecture trade-offs. The eICU dataset employed in the study has a restricted temporal coverage (only 2 years of data), which constrains the generalizability of time-dependent predictive outcomes to longer time horizons. Additionally, the Informer architecture—while optimized for long-term prediction tasks—exhibits a trade-off in that it has relatively lower sensitivity to short-term temporal clinical patterns (Zhou et al., 2021), which warrants cautious application of the model in clinical settings where short-window risk stratification is prioritized. For recurrent neural networks (RNNs), their predictive performance declined notably with extended time windows, whereas the other four models achieved favorable efficacy (AUROC >0.88) after incorporating long-cycle patient data. This discrepancy arises from inherent architectural constraints of RNNs: first, RNN training *via* Backpropagation Through Time (BPTT) may be susceptible to vanishing or exploding gradient issues in long sequences, which impairs the capture of long-term data dependencies; second, their sequential processing paradigm limits parallel computing capacity, further compromising long-term information retention (Chen et al., 2024). By contrast, the other four models integrate targeted architectural optimizations (*e.g.*, gating or attention mechanisms) that shorten information propagation paths and better preserve long-term temporal features (Al Olaimat & Bozdag, 2024; Zhou et al., 2023).

2. Data heterogeneity-related performance attenuation in external validation. A mild decline in AUROC was observed for all five base models and the stacked model in the external validation cohort, which is hypothesized to be associated with inherent data heterogeneity between the two datasets. This heterogeneity includes discrepancies in the distribution of patients’ baseline characteristics and vital signs, variations in ICD coding protocols and diagnostic criteria across medical centers, and differences in ICU monitoring equipment and clinical practice standards due to the retrospective, multicenter nature of data collection. Further, the two datasets differ substantially in temporal scope (MIMIC-IV covers nearly 10 years of data (2008–2019), while eICU only includes 2014–2015 records), which may have contributed to the performance fluctuation. Despite this, several models in the study still maintained satisfactory predictive efficacy on the heterogeneous external dataset. Moreover, minor data inconsistencies arising from ICD code version updates may have impacted model training and validation; while data alignment strategies were implemented to mitigate this issue, residual impacts could not be fully eliminated.

3. Model interpretability constraints. To process patient data with variable-length diagnostic records, the study adopted mask layer technology, which introduces a limitation in that the model’s internal path information is difficult to trace. This compromises the model’s overall interpretability, a key consideration for translational application in clinical decision-making scenarios.

Future research could focus on developing and applying deep learning (DL) models integrated with biochemical diagnostic indicators from each of patients’ ICU admissions, while also conducting multicenter prospective validation studies and enhancing model interpretability, to better inform clinical decision-making. The predictive capability of such deep learning models still requires further improvement (Wang & Hu, 2023).

## Conclusions

Deep learning models, including ensemble approaches, can predict in-hospital all-cause mortality in ICU patients using longitudinal diagnostic trajectories, admission diagnostic information, and demographic data. However, model performance varies across different external validation cohorts. While the stacking model demonstrated competitive overall performance across two independent external test sets (MIMIC-III and eICU), simpler architectures such as LSTM achieved the highest AUROC in the eICU cohort (0.802), whereas the Informer model—despite its theoretical advantages—showed relatively lower generalizability in external settings. Calibration analysis revealed some degree of miscalibration (*e.g.*, probability compression), indicating that model outputs should be interpreted with caution when applied to new populations. These findings suggest that deep learning-based risk stratification has potential utility in critical care, but further refinement—particularly in terms of calibration and cross-institution generalizability—is needed before clinical deployment. Model performance and calibration characteristics should be carefully evaluated in local settings prior to implementation.

## Supplemental Information

10.7717/peerj.21631/supp-1 Supplemental Information 1 Appendix.

10.7717/peerj.21631/supp-2 Supplemental Information 2 GRU Model.

10.7717/peerj.21631/supp-3 Supplemental Information 3 Transformer.

10.7717/peerj.21631/supp-4 Supplemental Information 4 RNN.

10.7717/peerj.21631/supp-5 Supplemental Information 5 lstm.

10.7717/peerj.21631/supp-6 Supplemental Information 6 Informer.

10.7717/peerj.21631/supp-7 Supplemental Information 7 Stacking.

## Additional Information and Declarations

### Data Availability

The following information was supplied regarding data availability: The code is available in the Supplemental Files. The MIMIC-IV data is available at Physionet: Johnson, A., Bulgarelli, L., Pollard, T., Gow, B., Moody, B., Horng, S., Celi, L. A., & Mark, R. (2024). MIMIC-IV (version 3.1). PhysioNet. RRID:SCR_007345. https://doi.org/10.13026/kpb9-mt58
The eICU data is available at: - https://eicu.mit.edu/
- Pollard, T., Johnson, A., Raffa, J., Celi, L. A., Badawi, O., & Mark, R. (2019). eICU Collaborative Research Database (version 2.0). PhysioNet. RRID:SCR_007345. https://doi.org/10.13026/C2WM1R
The MIMIC-III Clinical Database is available at: Johnson, A., Pollard, T., & Mark, R. (2016). MIMIC-III Clinical Database (version 1.4). PhysioNet. RRID:SCR_007345. https://doi.org/10.13026/C2XW26
