# Supplementary material for: Deep learning-based in-hospital mortality prediction using long-term sequential data in ICU patients: a multi-center validation study
Source: PeerJ. 2026 Aug 24;14:e21631. doi: 10.7717/peerj.21631 (PMC13525758; doi:10.7717/peerj.21631)
Supplement: Supplementary file 1 [file peerj-14-21631-s001.docx]

**Supplementary Data**

**1.Stacking Ensemble Model Architecture**

In this study, we implement a two–level stacking ensemble framework for in-hospital mortality prediction in intensive care unit (ICU) patients. The overall architecture comprises a base learner layer and a meta-learner layer, designed to leverage heterogeneous model architectures and capture complementary predictive patterns from longitudinal ICD code sequences and auxiliary demographic/clinical features.

**1.Base Learner Layer**
The base layer consists of five independently–trained deep neural network architectures, each tailored to capture distinct aspects of temporal and contextual information from patient data. All base learners take as input:
(a) an ICD code sequence for each ICU stay, encoded via a shared LabelEncoder vocabulary, and
(b) auxiliary static features including time interval between ICU admissions (TIME_DIFF), patient age, and gender one-hot encodings (gender_F, gender_M).

The five base learners are

**Informer:** Implements the ProbSparse self-attention mechanism and self-attention distilling strategy, enabling efficient modeling of ultra-long sequences. By selecting the most informative query vectors for attention computation, complexity is reduced from O(L^2^) to O(LlogL), while distillation reduces sequence length layer-by-layer, improving scalability.

**Transformer Encoder:** A standard Transformer-based encoder with multi-head self-attention and attention-based pooling, capable of capturing long-range dependencies without explicit recurrence.

**Bidirectional LSTM:** A gated recurrent architecture capable of modeling long-term dependencies in both forward and backward temporal directions.

**Bidirectional GRU:** A computationally lighter recurrent architecture that retains the gating mechanisms of LSTMs, effective for moderately long dependencies.

**Bidirectional RNN:** A simple recurrent structure capturing local sequential dynamics, serving as a lightweight complementary learner.

Each base learner produces a scalar probability pi∈(0,1), representing the estimated mortality risk for the given input. All parameters are learned independently; there is no parameter sharing across base learners.

**2. Meta-Learner Layer**
The meta-learner is designed to integrate the predictions of the base learners along with the auxiliary features, learning an adaptive fusion strategy. The input to the meta-learner is the concatenation of:

The vector of base learner predictions p=[p1,p2,p3,p4,p5]

The auxiliary feature vector $\mathbf{x}_{aux}\in{\mathbb{\mathbb{R}}}^{4}$The meta-learner employs a gated multi-layer perceptron (MLP) architecture:

Feature-wise gating:

Base prediction vector is passed through a bias–free linear transformation and a sigmoid activation to generate element–wise gating coefficients

$$\mathbf{g}_{p}=\sigma(W_{p}\mathbf{p}),\quad\mathbf{g}_{p}\in(0,1)^{5}$$

Auxiliary feature vector xaux\mathbf{x}_{aux} undergoes the same transformation, yielding

$$\mathbf{g}_{x}=\sigma(W_{x}\mathbf{x}_{aux}),\quad\mathbf{g}_{x}\in(0,1)^{4}$$

The transformed inputs are scaled element–wise:

$$\mathbf{p}'=\mathbf{g}_{p}\odot\mathbf{p},\quad\mathbf{x}'=\mathbf{g}_{x}\odot\mathbf{x}_{aux}$$

Concatenation and classification:The gated prediction and auxiliary feature vectors are concatenated into a single vector $[\mathbf{p}',\mathbf{x}']$.

This vector is fed into a two–layer MLP: a fully connected layer with ReLU activation, followed by dropout regularization, and a final fully connected layer with sigmoid activation producing the final mortality probability estimate $\hat{y}\in(0,1)$.The meta-learner is trained using binary cross–entropy loss with the area under the receiver operating characteristic curve (AUROC) as the primary performance metric.

**3. Training and Evaluation Protocol**
The ensemble training procedure follows a strict separation of datasets to prevent information leakage:

Base learner training: All five base models are trained on Dataset 1 using balanced mini-batches to mitigate class imbalance.

Meta-learner training: Trained base learners are applied to Dataset 2 to generate out-of-sample prediction vectors, which are combined with the corresponding auxiliary features to form the meta-learner’s training set. Dataset 2 is split into an 80% training and 20% validation subset for meta-learner optimization.

Final evaluation: The fully trained stacking model (base learners + meta-learner) is evaluated on the held–out Dataset 3. We report AUROC, Brier score, and produce both ROC curves and calibration plots to assess ranking performance and probability calibration.

This two–level stacking architecture offers several advantages:

Complementary representation learning: Different sequence models specialize in capturing different temporal and contextual patterns in patient trajectories.

Adaptive integration: The gated MLP meta-learner dynamically weights base learner outputs and auxiliary features according to the patient’s profile.

Robustness: Dataset partitioning ensures that the meta-learner is trained on predictions from unseen patient records, mitigating overfitting to base learner idiosyncrasies.

As a result, the proposed stacking ensemble achieves improved predictive performance and stability over any single constituent model.

**2.Introduction to the Informer model architecture：**

1)Self-attention mechanism: from fully connected to sparsified

Transformer: Utilizing the standard Scaled Dot-Product Attention, the computational complexity is $\text{O}\text{(}\text{L}^{\text{2}}\text{)}$ ( L represents the sequence length). Each query needs to compute the dot product with all keys, leading to an explosion in computational/memory overhead for long sequences.

INFORMER: Proposes ProbSparse self-attention, optimized through the following methods:

Perform sparsity evaluation on query (such as the difference between Log-Sum-Exp and arithmetic mean), and select the subset of queries that contribute the most to prediction;

By only calculating the dot product between this subset and the key, the complexity is reduced to $\text{O}\text{(}\text{L}\log\text{L}\text{)}$;

Maintain the ability to capture long-distance dependencies comparable to standard self-attention.

2)Encoder: From stacking bottleneck to distillation compression

Transformer: After stacking multiple layers of encoders, the total memory complexity is $\text{O}\text{(}\text{J}\text{⋅}\text{L}^{\text{2}}\text{)}$ ( J represents the number of layers). When dealing with long sequence inputs, memory consumption grows exponentially with both the number of layers and sequence length, severely limiting scalability.

INFORMER: Introducing Self-Attention Distilling:

Each layer reduces the length of the input sequence by half through convolution and pooling, highlighting the dominant attention features;

The total space complexity is reduced to $\text{O}\text{((2−}\text{ϵ}\text{)}\text{L}\log\text{L}\text{)}$ ( $\text{ϵ}$ is the distillation rate), supporting extremely long sequence inputs L>10^4.

3)Decoder: From dynamic decoding to one-step generation

Transformer: Using dynamic decoding, it requires generating predictions step by step (predicting step \(t+1\) relies on the result of step \(t\)). This results in slow inference speed and is prone to accumulating errors.

INFORMER: Designing a Generative Decoder:

During training, the target sequence (including future time steps) is padded with zeros as input, and the model directly learns the mapping from "input - future";

During inference, the entire long sequence output is generated in one step, avoiding the delay and error propagation of step-by-step decoding, resulting in a significant speedup.[1]

**3.Top 20 ranking of patient disease frequency in the dataset**

2-a

2-b

2-c

For other variables, especially regarding the diagnosis of patient diseases and the ICD coding record format, the recording standards of MIMIC and eICU medical systems differ, and the ICD coding format has been updated from the original ICD-9 to ICD-10 during this period, which can lead to heterogeneity among some datasets. We minimize the data errors caused by coding heterogeneity by synonymizing ICD-10 codes to the more proportion ICD-9 codes. According to the statistics of participant diagnoses in the three datasets, the top three most frequently diagnosed diseases in MIMIC-IV are acute kidney failure (1.50%), essential hypertension (1.26%), and congestive heart failure (1.08%); in MIMIC-III, the top three are essential hypertension (2.67%), congestive heart failure (2.50%), and atrial fibrillation (1.97%); in eICU, the top three are acute respiratory failure (6.95%), acute kidney failure (4.02%), and pneumonia (3.37%). Considering the number of cases, AKI, essential hypertension, and ARF account for the top three diagnoses among all ICU patients throughout their entire hospitalization period.

**4.Inclusion and exclusion criteria for different time periods on the AUC curve**

 3-a

3-b

3-c

3-d

3-e

3-f

Figures1. 3-a to 3-e show the differences in performance of five deep learning models on different datasets before and after the inclusion criteria were applied. Figure 3-f presents the AUC changes of the five models in a line chart.

| Models | 2years | 3years | 5years | all years | Random guess |
| --- | --- | --- | --- | --- | --- |
| INFORMER | 0.904 | 0.918 | 0.945 | 0.949 | 0.500 |
| Transformer | 0.896 | 0.892 | 0.909 | 0.928 | 0.500 |
| LSTM | 0.894 | 0.919 | 0.882 | 0.920 | 0.500 |
| RNN | 0.886 | 0.828 | 0.797 | 0.771 | 0.500 |
| GRU | 0.908 | 0.912 | 0.908 | 0.911 | 0.500 |

We can observe that, except for the significant decline in AUC of RNN as the time period increases, all other models exhibit an upward trend, which is consistent with the viewpoint discussed in the main text. This is because the model architecture of RNN determines its inability to effectively retain useful information in long-period sequence data, leading to a gradual decrease in its AUC.[2]

**5.Schematic diagram of prediction model usage**

We input the diagnosis information of patients into the model, then calculate their survival probability, and remind the medical staff to strengthen their care, so as to reduce their risk of death

**6，Model Performance Analysis Based on AUPRC and F1 Metrics**

Summary of Model AUPRC and F1 Scores

|  | Data base | LSTM | Transformer | RNN | GRU | Informer | Stacked model |
| --- | --- | --- | --- | --- | --- | --- | --- |
| AUPRC |  |  |  |  |  |  |  |
|  | MIMIC-IV | 0.8965 | 0.9013 | 0.8912 | 0.9441 | 0.9069 | \ |
|  | MIMIC-III | 0.8315 | 0.8297 | 0.8200 | 0.6509 | 0.8543 | 0.8624 |
|  | eICU | 0.6891 | 0.7143 | 0.6939 | 0.7167 | 0.6063 | 0.8211 |
| F1 |  |  |  |  |  |  |  |
|  | MIMIC-IV | 0.8274 | 0.8106 | 0.8122 | 0.8605 | 0.8282 | \ |
|  | MIMIC-III | 0.7619 | 0.7496 | 0.7161 | 0.3321 | 0.7819 | 0.8017 |
|  | eICU | 0.6453 | 0.6803 | 0.5438 | 0.2017 | 0.6514 | 0.6959 |

Six models (LSTM, Transformer, RNN, GRU, Informer, and Stacked model) were evaluated on three clinical databases (MIMIC-IV, MIMIC-III, and eICU), with their performance quantified by the Area Under the Precision-Recall Curve (AUPRC) and F1 score. AUPRC is particularly robust for imbalanced clinical datasets, while the F1 score balances precision and recall, both of which are crucial for assessing the reliability of models in clinical prediction tasks. The following analysis focuses on the performance of each individual model across the three databases, strictly based on the provided table data with all metrics fully supplemented.

The LSTM model exhibited stable performance across the three databases, with complete metrics available for all datasets. In terms of AUPRC, it achieved 0.8965 on MIMIC-IV, 0.8315 on MIMIC-III, and 0.6891 on eICU, showing a clear downward trend from MIMIC-IV to eICU. For the F1 score, the LSTM model obtained 0.8274 on MIMIC-IV, 0.7619 on MIMIC-III, and 0.6453 on eICU, which was consistent with its AUPRC performance. This indicates that the LSTM model has good adaptability to the MIMIC-series databases but relatively poor performance on the eICU dataset, possibly due to higher data heterogeneity in eICU.

The Transformer model showed steady performance with complete metrics across all databases. Its AUPRC values were 0.9013 (MIMIC-IV), 0.8297 (MIMIC-III), and 0.7143 (eICU), with the highest performance on MIMIC-IV and the lowest on eICU. Correspondingly, its F1 scores were 0.8106 (MIMIC-IV), 0.7496 (MIMIC-III), and 0.6803 (eICU). Compared with the LSTM model, the Transformer model achieved a higher AUPRC and F1 score on eICU, demonstrating its better ability to capture complex temporal features in heterogeneous datasets.

The RNN model displayed moderate and relatively stable performance across all three databases, with no missing metrics. Its AUPRC values were 0.8912 (MIMIC-IV), 0.8200 (MIMIC-III), and 0.6939 (eICU), maintaining a consistent downward trend across datasets. For the F1 score, the RNN model reached 0.8122 on MIMIC-IV, 0.7161 on MIMIC-III, and 0.5438 on eICU. Notably, it achieved the highest F1 score among all models on MIMIC-IV but experienced a significant performance drop on eICU, second only to the GRU model.

The GRU model performed the worst among all models, with complete but dramatically fluctuating metrics across databases. Its AUPRC values were 0.9441 (MIMIC-IV), 0.6509 (MIMIC-III), and 0.7167 (eICU), showing an extreme decline on MIMIC-III. The F1 score of the GRU model was even more unsatisfactory: 0.8605 on MIMIC-IV, 0.3321 on MIMIC-III, and only 0.2017 on eICU. This significant performance fluctuation indicates that the GRU model has poor adaptability to heterogeneous clinical datasets and is not suitable for cross-database clinical prediction tasks.

The Informer model showed excellent performance on the MIMIC-series databases but poor performance on eICU, with complete metrics for all datasets. Its AUPRC values were 0.9069 (MIMIC-IV), 0.8543 (MIMIC-III), and 0.6063 (eICU), ranking second among all models on MIMIC-IV and MIMIC-III. Its F1 scores were 0.8605 (MIMIC-IV), 0.7819 (MIMIC-III), and 0.6514 (eICU), reflecting its strength in capturing temporal dependencies in the MIMIC databases but weakness in handling the high-noise and heterogeneous data in eICU.

The Stacked model outperformed other models on MIMIC-III and eICU, with superior metrics in both AUPRC and F1 score (no data available for MIMIC-IV). Its AUPRC values were the highest on MIMIC-III (0.8624) and eICU (0.8211). Similarly, its F1 scores were 0.8017 (MIMIC-III) and 0.6959 (eICU), maintaining a consistent leading position on these two databases. This highlights the advantage of the integrated architecture, which integrates the strengths of different base models and thus has strong adaptability to diverse clinical datasets (excluding MIMIC-IV due to missing data).

In summary, all models demonstrated better performance on MIMIC-IV and MIMIC-III than on eICU, which may be attributed to differences in data volume, feature completeness, and patient population characteristics among the three databases. The Stacked model exhibited strong performance and adaptability on MIMIC-III and eICU (with no MIMIC-IV data), while the GRU model showed the worst stability and adaptability. These findings provide valuable guidance for the selection and optimization of models in clinical outcome prediction tasks targeting different databases.

**7,Model Hyperparameter Grid Search Range**

| **Model name** | **Learning rate** | **Weight decay** | **embed_dim** | **hidden_dim** | **Num layers** |
| --- | --- | --- | --- | --- | --- |
| GRU | [0.0001, 0.001, 0.01] | [0.0001, 0.001, 0.005, 0.01] | [32, 64, 128] | [32, 64, 128] | [1, 2, 3] |
| LSTM | [0.0001, 0.001, 0.01] | [0.0001, 0.001, 0.005, 0.01] | [32, 64, 128] | [32, 64, 128] | [1, 2, 3] |
| RNN | [0.0001, 0.001, 0.01] | [0.0001, 0.001, 0.005, 0.01] | [32, 64, 128] | [32, 64, 128] | [1, 2,3] |
| Transformer | [1e-5, 5e-5, 1e-4, 5e-4] | [1e-4, 1e-3, 5e-3] | [64, 128, 256] | [128, 256, 512] | [2, 3, 4] |
| Informer | [1e-5, 5e-5, 1e-4, 5e-4] | [1e-4, 1e-3, 5e-3] | [64, 128, 256] | [128, 256, 512] | [2, 3, 4] |

| Stacked model | Grid search |
| --- | --- |
| lr_informer | [1e-4, 5e-4, 1e-3, 2e-3] |
| lr_transformer | [1e-4, 5e-4, 1e-3, 5e-3] |
| lr_lstm / lr_gru / lr_rnn | [1e-4, 5e-4, 1e-3] |
| lr_meta | [1e-4, 5e-4, 1e-3, 2e-3] |
| batch_size | [16, 32, 64,128] |
| weight_decay | [1e-5, 1e-4, 1e-3] |
| meta_hidden_dim | [64, 128, 256,512] |

REFERENCES:

1.Zhou H, Zhang S, Peng J, et al. Informer: Beyond Efficient Transformer for Long Sequence Time-Series Forecasting. Proceedings of the AAAI Conference on Artificial Intelligence. 2021 May 18;35(12):11106-11115. doi: 10.1609/aaai.v35i12.17325.

2.Chen Y, Zhang X, Sun M. State Explosion in RNN-Based Long-Context Modeling: Mechanism and Mitigation. OpenReview. 2024;12121. doi:10.48550/arXiv.2409.28763.
